# A high mobility air-stable n-type organic small molecule semiconductor with high UV–visible-to-NIR photoresponse

**DOI:** 10.1038/s41377-022-00936-z

**Published:** 2022-07-27

**Authors:** Ying-Shi Guan, Jing Qiao, Yingying Liang, Hari Krishna Bisoyi, Chao Wang, Wei Xu, Daoben Zhu, Quan Li

**Affiliations:** 1 Institute of Advanced Materials and School of Chemistry and Chemical Engineering, Southeast University, 211189 Nanjing, China; 2 Beijing National Laboratory for Molecular Sciences, Key Laboratory of Organic Solids Institute of Chemistry, Chinese Academy of Sciences, 100190 Beijing, China; 3 Advanced Materials and Liquid Crystal Institute and Chemical Physics Interdisciplinary Program, Kent State University, Kent, OH 44242 USA

**Keywords:** Optical materials and structures, Optical sensors

## Abstract

An organic semiconductor with high carrier mobility and efficient light absorption over a wide spectral range is of the most important yet challenging material for constructing a broadband responsive organic photodetector. However, the development of such organic semiconductors, especially for air-stable n-type organic small molecule semiconductors, is still at an early stage. Here we report the fabrication of high-performance n-type semiconducting crystalline nanosheets and the development of air-stable field-effect transistors, phototransistors, with high response over a broad spectrum. The n-type small molecule semiconductor is assembled into a crystalline nanosheet based on the solvent-phase interfacial self-assembly method. N-type field-effect transistors with high electron mobility are fabricated and their electrical performances exhibit excellent air stability. Impressively, the demonstrated phototransistors exhibit an ultrahigh responsivity over a wide spectral range from 365 to 940 nm, with a maximum photoresponsivity of 9.2 × 10^5^ A W^−1^ and specific detectivity of 5.26 × 10^13^ Jones, which is the best performance among the reported n-type organic small molecule-based phototransistors.

## Abstract (web summary)

A broadband organic phototransistor is achieved based on an air stable n-type organic semiconductor, which exhibits ultrahigh photoresponsivity in a wide wavelength range from the UV, visible to the NIR.

## Graphical abstract

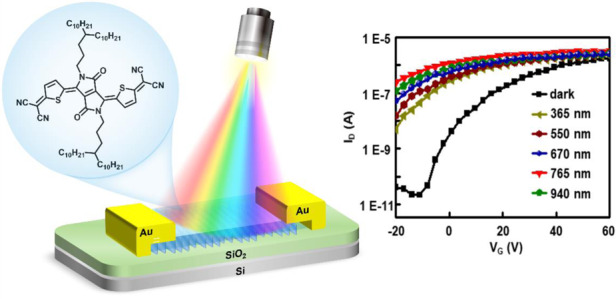

## Introduction

Light detection in a broad wavelength range from the ultraviolet (UV), visible to the near infrared (NIR) is of use in many applications such as environmental monitoring, imaging sensors, biomedical application, and industrial process control^1–4^. It is desirable to create a broadband responsive photodetector^5^. Such photodetectors are, however, technologically challenging to build. To create a photodetector, the photo-active semiconductor is an indispensable material which dominates the performance of photodetector^6–8^. The high carrier mobility semiconductors with efficient light absorption over a wide spectral range, are particularly crucial to realize the high-performance broadband photodetectors. Organic semiconductors are promising candidates for light detection because of their low-cost fabrication, tunable optoelectronic properties, and good solution processibility^9–11^.

Recent efforts in the design of molecular materials and device architectures have resulted in extraordinary achievement in the performance of organic phototransistors based on organic semiconductors^10–12^. A variety of organic semiconducting materials including organic small molecules and polymers have been reported for the application of organic phototransistors (OPTs) to detect UV–visible light^1,13,14^. Despite the huge progress, OPTs with a response spectrum covering a broad spectral range from UV to NIR are still rare especially the OPTs based on a single active component. In the majority of the cases, the broadband OPTs are achieved through mixing two or more photo-active materials with different light absorption regions such as donor/acceptor bulk heterojunction (BHJ) structure^15^, due to the limited absorption in a single material, which is the most difficult issues to solve for realizing the light sensing from the UV–visible to the NIR region in single material based organic phototransistor^16^. Compared to bilayers or bulk heterojunction blends, organic phototransistors based on a single component are considered to be ideal candidates for their low-cost and simple fabrication^17^. In addition, OPTs fabricated from donor/acceptor two components active layers often exhibit ambipolar charge transport characteristics, which usually reduce the detectivity^18^. It would be advantageous to have a single component-based OPTs system with high photoresponsivity spanning the full range from the UV, visible to the NIR. However, due to lack of applicable organic semiconducting materials which can absorb incident light efficiently over a wide range of wavelengths as well as having high carrier mobility, the development of organic broadband phototransistor based on a single component active layer is largely restricted.

**Fig. 1:**
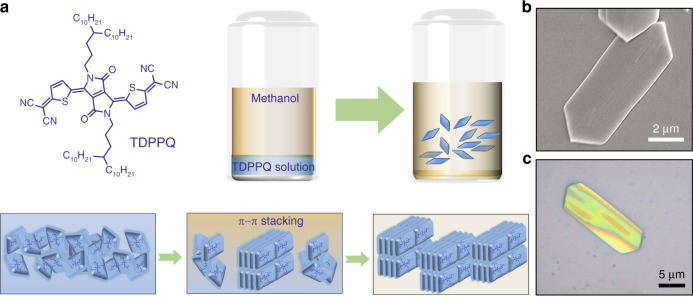
The fabrication and characterization of the assembled TDPPQ nanosheet. **a** The chemical structure of the TDPPQ and schematic illustration of the solvent-phase interfacial self-assembly method to grow TDPPQ nanosheets. **b** The typical SEM image of the TDPPQ nanosheet fabricated from 0.1 mg/mL TDPPQ solution. **c** The typical optical image of the TDPPQ nanosheet fabricated from 0.1 mg/mL TDPPQ solution

Here we report a solution-processed air-stable n-type small molecule-based organic phototransistor as a broadband photodetector which exhibits ultrahigh photoresponsivity in a wide wavelength range from the UV, visible to the NIR. The phototransistor uses n-type organic small molecule, thiophene-diketo-pyrrolopyrrole-based quinoidal (TDPPQ) (Fig. 1a), as the photo-active layer. Especially, the n-type TDPPQ molecules can be self-assembled into crystalline nanosheets based on solvent-phase interfacial self-assembly method^19^, which particularly leads to a highly ordered packing structure of TDPPQ molecules and the resulting high field-effect electron mobility. The TDPPQ crystalline nanosheets-based organic transistors exhibit a maximum field-effect electron mobility (µ_FE_) of 2.1 cm^2^ V^−1^ s^−1^. In addition, the transistor devices show excellent air stability under ambient conditions and their field-effect electron mobility values only have a very slight change after 2 months of storage in air. More importantly, our devices exhibit a wide spectral response covering from UV (365 nm) to NIR (940 nm). The maximum photoresponsivity and specific detectivity at a wavelength of 760 nm can reach up to 9.2 × 10^5 ^A W^−1^ and 5.26 × 10^13^ Jones, respectively.

## Results

As a well-known molecular building block, diketopyrrolopyrrole (DPP) has been successfully used in the construction of a large number of high-performance organic semiconductor materials because of its planar skeleton, electron-deficient nature and ability to form hydrogen bonds, which would lead to strong π − π stacking interactions and large intramolecular charge transfer facilitating charge transport in organic semiconductor materials^20–22^. In this work, we fabricate crystalline TDPPQ nanosheets using the solvent-phase interfacial self-assembly method. The schematic illustration of the crystalline TDPPQ nanosheets fabrication based on such an assembly method is shown in Fig. 1a. Especially, by dissolving TDPPQ in chloroform in a screw-type vial, the TDPPQ solution is formed. Subsequently, a bad solvent (methanol) is added very slowly onto the surface of the chloroform solution drop by drop using a syringe. Because of the significantly lower density of methanol (0.791 g/cm³) compared with the chloroform (1.48 g/cm³), the methanol stays in the upper layer and the TDPPQ solution is in the bottom layer. After several hours’ standing, the methanol slowly diffuses into the TDPPQ solution phase, inducing the supramolecular self-assembly of TDPPQ molecules. During the two-phase diffusion process, TDPPQ molecules at the interface between methanol and chloroform start to crystallize, which could effectively self-assemble into well-ordered nanosheets through strong π−π stacking interactions between the TDPPQ molecules. The TDPPQ nanosheets can be obtained and transferred onto various substrates for further study. The TDPPQ solution with different concentrations is prepared, and the morphologies of the supramolecular assemblies are examined. It is noted that the TDPPQ supramolecular assemblies tend to aggregate together at a high concentration TDPPQ solution. Figures 1b and S1 display a set of scanning electron microscopy (SEM) images of TDPPQ supramolecular assemblies fabricated from TDPPQ solution with different concentrations. The TDPPQ nanosheets exhibit regular hexagonal shape with the size ranging from several micrometers to dozens of micrometers in length. The TDPPQ supramolecular assemblies at a relatively lower concentration TDPPQ solution exhibit a single nanosheet which is convenient for electronic device fabrication (Fig. 1c).

**Fig. 2:**
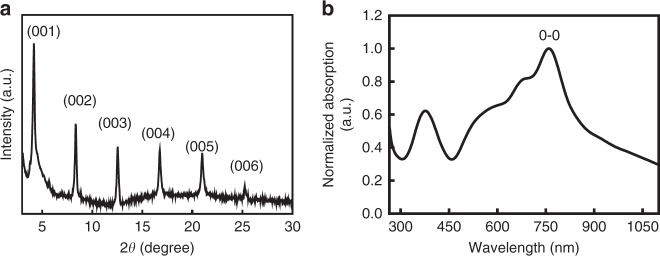
The characterization of the assembled TDPPQ nanosheet. **a** The XRD pattern of the TDPPQ nanosheets fabricated from 0.1 mg/mL TDPPQ solution. **b** The absorption spectrum of the TDPPQ nanosheets dispersed in methanol

A typical transmission electron microscope (TEM) image of a TDPPQ nanosheet and its corresponding selected area electron diffraction (SAED) pattern are shown in Fig. S2. The TEM image of the nanosheet further confirms the hexagonal morphology. X-ray diffraction (XRD) spectroscopy is employed to gain insight into the molecular packing within the nanosheets. As shown in Fig. 2a, six strong distinct multiorder diffraction peaks at 2*θ* = 4.17°, 8.34°, 12.55°, 16.75°, 20.98° and 25.23°, observed in the XRD patterns, further demonstrate that a highly ordered packing structure exists within the TDPPQ nanosheets. The multiorder diffraction peaks correspond to Bragg diffraction of the (001), (002), (003), (004), (005), and (006) planes revealing the layer-by-layer growth of the TDPPQ molecules, as shown in Fig. S3. The lamella structure of the nanosheets is further demonstrated by the SEM images (Fig. S4) of the growth process for the nanosheets. Interestingly, the absorption spectrum of the nanosheets dispersed in methanol is broadened relative to the corresponding TDPPQ thin film spectrum, and the 0–0 vibrational peak is red-shifted by 16 nm, showing a broad absorption from ultraviolet (250 nm) to near infrared (1100 nm) with the maximum absorption wavelength (0–0 vibrational peak) at 760 nm (Fig. 2b)^23^. This result indicates that a highly ordered molecular packing structure exists within the TDPPQ nanosheet, which is consistent with the XRD result. That’s to say, a very broad absorption can be achieved in one single organic semiconductor. The unique absorption spectrum of the TDPPQ nanosheet enables its potential application in organic broadband photodetector which can detect light over a wide range of wavelengths covering from the UV, visible to the NIR.

**Fig. 3:**
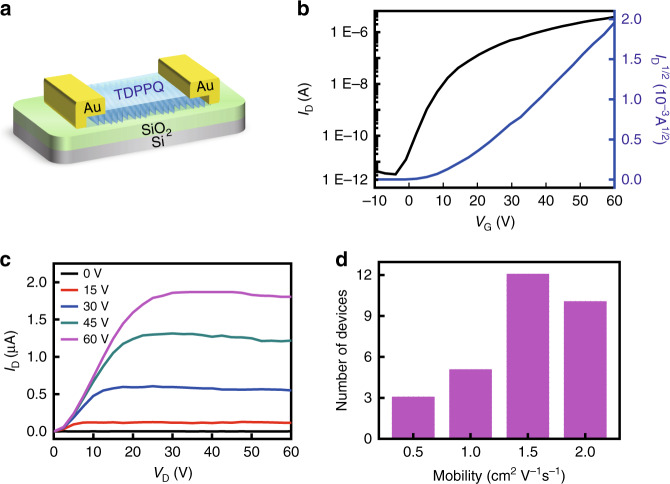
The self-assembled TDPPQ-nanosheet-based organic field-effect transistor. **a** The schematic illustration of the TDPPQ-nanosheet-based top-contact bottom-gate organic transistor. **b** Transfer characteristic of a representative device. **c** Output characteristic of a representative device. **d** Mobility distribution of the TDPPQ-nanosheet-based transistors

The electrical performance of the crystalline TDPPQ nanosheet is investigated based on field-effect transistors. The devices adopt bottom-gate and top-contact configuration using n-doped silicon wafer as the gate electrode, OTS-modified SiO_2_ layer as the dielectric layer and gold electrodes as the source and drain electrodes. The schematic diagram of the transistor is shown in Fig. 3a. To fabricate the transistors, organic ribbon is used as soft shadow mask. The detailed device fabrication steps are presented in supporting information and the schematic illustration of the device fabrication procedure are displayed in Fig. S5. All the measurements are performed under ambient conditions. The representative transfer curve and output curve of a typical transistor device are displayed in Fig. 3b, c, respectively. The drain current (*I*_D_) of the transfer curve shows a significant increase when the gate voltage is swept from −10 V to 60 V at a fixed drain voltage, demonstrating unipolar n-type transport characteristics. Drain currents (*I*_D_) from the output curves saturate when the drain voltage (*V*_D_) is bigger than 30 V at the measured gate voltages (*V*_G_).

The electron mobility of the crystalline TDPPQ-nanosheet-based field-effect transistor can be calculated from the saturation regime of the transfer curve. In total, 30 devices are tested, and the calculated average field-effect mobility is 1.5 cm^2^ V^−1^ s^−1^ (Fig. 3d). It seems that the size of the nanosheet has an effect on the transistor performance. As shown in Fig. S6, the crystalline TDPPQ nanosheets with a small width exhibit much higher mobility values. For the best device, the electron mobility can reach up to 2.1 cm^2^ V^−1^ s^−1^. Benefiting from the highly ordered packing structure within the nanosheet, the crystalline TDPPQ nanosheet has a much higher carrier mobility than that of the spin-coated TDPPQ thin film^23^. Generally, the high carrier mobility organic semiconductors require a dense molecular packing structure, which ensures the effective overlap of the π-orbitals. The high electron mobility of the TDPPQ nanosheet can be attributed to the highly ordered molecular packing structure enabling the long-range order, which can form a continuous conductive network. The aligned TDPPQ molecules with enhanced electronic coupling between neighboring molecules can form efficient charge carrier channel to facilitate the electron transport. In the spin-coated TDPPQ thin film, there are many grain boundaries impeding the electron coupling and eliminating charge transfer. Therefore, the assembled TDPPQ nanosheet exhibits a much higher mobility. More importantly, the TDPPQ-nanosheet-based transistor devices show excellent air stability under ambient conditions. The devices are stored in air and remeasured. A typical evolution of the electrical performance of the device versus storage time is shown in Fig. S7. Because the lowest unoccupied molecular orbital (LUMO) energy level of the TDPPQ is −4.51 eV^23^, lower than −4.0 eV, the mobility of the TDPPQ-nanosheet-based transistors experience a slight decrease after 2 months of storage in air. Owing to the high electron mobility and excellent air stability, the crystalline TDPPQ would be a very promising n-type organic semiconductor for practical application. However, the assembled TDPPQ nanosheets could have some limitation on scalable fabrication, which would limit their further application in the transistor array. We are currently exploring other approaches such as solution shearing, screen printing and drop casting to create two-dimensional (2D) crystalline thin films of organic semiconductors. Such 2D crystalline TDPPQ thin films would be the potential candidate for the fabrication of transistor array in high throughout in the future.

**Fig. 4:**
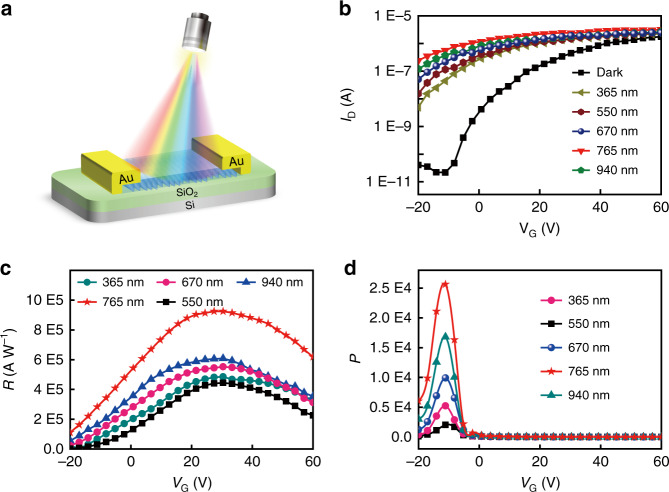
The phototransistor based on the self-assembled TDPPQ nanosheet. **a** The schematic illustration of the TDPPQ-nanosheet-based phototransistor. **b** Photocurrent responses of the TDPPQ-nanosheet-based phototransistor under the illumination of different monochromatic light (365 nm, 550 nm, 670 nm, 765 nm, and 940 nm) with a fixed light intensity of 600 μW cm^−2^. **c** Photoresponsivity and **d** photosensitivity of a representative device under the illumination of different monochromatic light (365 nm, 550 nm, 670 nm, 765 nm, and 940 nm) with a fixed light intensity of 600 μW cm^−2^

Subsequently, the photoresponse behaviors of the crystalline TDPPQ-nanosheet-based phototransistor are explored under the illumination of monochromatic light with tunable wavelength and intensity. Figure 4a shows the schematic diagram of the phototransistor. The typical transfer curves of the fabricated phototransistor tested in dark and under the illumination of different monochromatic light (365 nm, 550 nm, 670 nm, 765 nm and 940 nm) with a fixed light intensity of 600 μW cm^−2^ are shown in Fig. 4b. A significant increase of the drain current can be observed under the illumination of different monochromatic light compared to the drain current in dark, indicating a very high sensitivity to monochromatic light of different wavelength. Generally, the photoresponsivity (*R*), and the *I*_light_/*I*_dark_ ratio (photosensitivity, *P*) are the key parameters for evaluating the performance of phototransistors in optoelectronic devices. Photoresponsivity (*R*), which exhibits how efficiently the phototransistors respond to the external light irradiation, can be defined as the following equation:

$$R = \left( {I_{{\rm{light}}} - I_{{\rm{dark}}}} \right)/SP_{{\rm{in}}}$$

where *I*_light_ is the drain current under light illumination, *I*_dark_ is the drain current in the dark, *S* is the effective channel area, and *P*_in_ is the incident optical power per unit area of the device.

In Fig. 4c, d, the representative photoresponsivity (*R*) and the photosensitivity (*P*) are plotted versus gate voltage (*V*_*G*_) for the crystalline TDPPQ-nanosheet-based phototransistors illuminated upon different monochromatic light. According to the equation, the phototransistors show extremely high photoresponsivity over a wide spectral range. The photoresponsivity are 4.0 × 10^5 ^A W^−1^, 3.1 × 10^5 ^A W^−1^, 4.8 × 10^5 ^A W^−1^, 9.2 × 10^5 ^A W^−1^, and 5.2 × 10^5 ^A W^−1^ under monochromatic light illumination of 365 nm, 550 nm, 670 nm, 765 nm, and 940 nm, respectively (Fig. S8), and the corresponding photosensitivity (*P*) are 5.2 × 10^3^, 2.0 × 10^3^, 9.9 × 10^3^, 2.5 × 10^4^, and 1.7 × 10^4^, respectively (Fig. S9). The maximum value of *R* can reach up to 9.2 × 10^5 ^A W^−1^ at *V*_G_ = 30 V with a maximum *P* value of 2.5 × 10^4^ at *V*_*G*_ = 11 V illuminated by 765 nm monochromatic light source, which is among the best performance of the reported organic semiconductor-based NIR phototransistors^24–28^. The comparison of the NIR phototransistor based on organic semiconductors is shown in Table S1 in Supporting Information. The photoresponsivity and photosensitivity at different wavelengths follow approximately the absorption spectrum of the crystalline TDPPQ nanosheets.

**Fig. 5:**
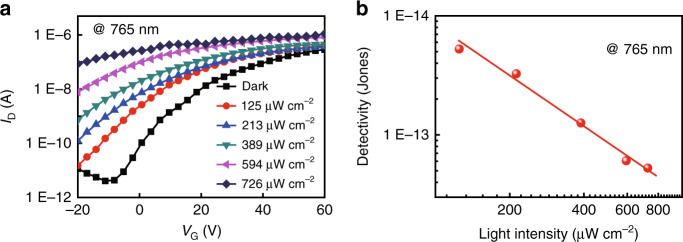
The detectivity of the phototransistor. **a** Photocurrent responses of the TDPPQ-nanosheet-based phototransistor under 765 nm monochromatic light illumination with different light intensity. **b** Detectivity of a representative device under 765 nm monochromatic light illumination

Another key parameter for evaluating the performance of the phototransistor is detectivity (D*), which can distinguish light signal from the background noise. D* can be calculated from the following equation:

$$D^ \ast = RS^{1/2}/\left( {2qI_{\rm{D}}} \right)^{1/2}$$

where *R* is the photoresponsivity value, *S* is the effective area of the phototransistor, *q* is the unit of charge and *I*_*D*_ is the dark current. The transfer curves of the phototransistor are further remeasured under 765 nm monochromatic light with various light intensity, which are shown in Fig. 5a. An obvious increase of the drain current is observed as increasing the light intensities. Figure 5b shows the D* of the phototransistor based on a single-crystalline nanosheet as a function of the monochromatic light intensity. The maximal D* of 5.26 × 10^13^ Jones can be achieved at an illumination light intensity of 125 μW cm^−2^, making the crystalline TDPPQ-nanosheet-based organic phototransistor comparable to or even better than previously reported photodetectors fabricated from inorganic materials^29–32^.

## Discussion

In conclusion, we have fabricated n-type crystalline TDPPQ nanosheets using a solution-processed solvent-phase interfacial self-assembly method, which leads to a highly ordered molecular packing structure within the TDPPQ nanosheets. The fabricated crystalline TDPPQ-nanosheet-based field-effect transistor devices exhibit a very high electron mobility and have excellent air stability. The unique wide spectral absorption of the crystalline TDPPQ nanosheets covering from ultraviolet (250 nm) to near infrared (1100 nm) endows their application in the organic broadband photodetector. The broadband phototransistors based on n-type organic small molecular crystalline TDPPQ nanosheets have been successfully fabricated. Impressively, the demonstrated organic phototransistors show an ultrahigh responsivity over a wide spectral range from 365 to 940 nm, with a maximum photoresponsivity of 9.2 × 10^5 ^A W^−1^ and specific detectivity of 5.26 × 10^13^ Jones, to the best of our knowledge, which is among the best performance of the reported n-type organic small molecule-based phototransistors. Our results demonstrated in this work provide promising possibilities for the construction of high-performance organic broadband photodetector and have great potential in solution-processed low-cost organic electronics and beyond.

## Materials and methods

Chemical reagents are purchased from Sigma-Aldrich, Acros, or Alfa Aesar and used without further purification. The TDPPQ is synthesized following our previously published procedure. Absorption spectra are recorded on a JASCO V-570 UV−vis spectrometer. One-dimensional grazing-incidence X-ray diffraction (1D-GIXRD) experiments are recorded on an Empyrean X-ray diffractometer equipped with a Pixcel 3D detector by using Cu Kα radiation. Electrical transport properties are measured under ambient conditions by using a semiconductor analyzer (Keithley 4200). The monochromatic light of 365 nm, 550 nm, 670 nm, 765 nm, and 940 nm is obtained from CEL-S500 Xenon Lamp Source by choosing an optical filter with different wavelengths. The half-width at half-maximum (HWHM) of the optical filters is 15–25 nm.

### Fabrication of the assembled single-crystalline nanosheet

1 mL TDPPQ solution in chloroform with different concentrations (5 mg/mL, 1 mg/mL and 0.1 mg/mL) is prepared firstly in a screw-type vial, then 5 mL methanol is added very slowly onto the surface of the chloroform solution drop by drop using a syringe. After 5–8 h’s standing, single-crystalline nanosheets can be obtained due to the strong π−π stacking interaction between the TDPPQ molecules.

### Substrate cleaning

Bare Si/SiO_2_ substrate is cut into 1 cm × 1 cm small square pieces, and then successively cleaned with pure water, piranha solution (H_2_SO_4_:H_2_O_2_ = 2:1), pure water, and pure isopropanol. The OTS-modified Si/SiO_2_ substrate is carried out by vapor deposition method. The cleaned Si/SiO_2_ small square pieces are dried at 90 °C for 0.5 h in the vacuum oven. After cooling to room temperature, 50 μL of OTS is placed on the wafers. Subsequently, this system is heated to 120 °C and maintained for 2 h under vacuum.

### Organic field-effect transistors (OFETs) device fabrication

Single-crystallined-nanosheet-based OFETs are constructed with a bottom-gate top-contact configuration, using a n-doped silicon wafer as the gate electrode, Au electrodes as the source and drain electrodes, and OTS-modified SiO_2_ as the dielectric layer. The detailed device fabrication is presented in Fig. S5. Firstly, a single-crystallined nanosheet of TDPPQ is deposited on an OTS/SiO_2_/Si substrate by drop-casting the dilute solution as above mentioned. Secondly, an organic ribbon is transferred onto the top surface of the nanosheet as a shadow mask. Thirdly, Au drain and source electrodes (~50 nm-thick) are deposited by thermal evaporation. Finally, the organic ribbon mask is peeled off to complete the device fabrication.

## Supplementary information

Supplementary Information
