# Supplementary material for: A high mobility air-stable n-type organic small molecule semiconductor with high UV–visible-to-NIR photoresponse
Source: Light Sci Appl. 2022 Jul 27;11:236. doi: 10.1038/s41377-022-00936-z (PMC9329299; doi:10.1038/s41377-022-00936-z)
Supplement: Supplementary file 1 — Supplementary Information [file 41377_2022_936_MOESM1_ESM.pdf]

Supplementary Information

A high mobility air stable n-type organic small molecule semiconductor with high UV-Visible-to-NIR photoresponse

Ying-Shi Guan[#], Jing Qiao[#], Yingying Liang, Hari Krishna Bisoyi, Chao Wang, Wei Xu[*], Daoben Zhu, Quan Li[*]

Dr. Y.-S. Guan, Dr. J. Qiao, Prof. Q. Li.
Institute of Advanced Materials and School of Chemistry and Chemical Engineering
Southeast University
Nanjing 211189, China
E-mail: quanli3273@gmail.com
Dr. Y. Liang, Dr. C. Wang, Prof. W. Xu, Prof. D. Zhu
Beijing National Laboratory for Molecular Sciences, Key Laboratory of Organic Solids
Institute of Chemistry, Chinese Academy of Sciences
Beijing 100190, China
E-mail: wxu@iccas.ac.cn
Dr. H. K. Bisoyi, Prof. Q. Li.
Advanced Materials and Liquid Crystal Institute and Chemical Physics Interdisciplinary Program
Kent State University
Kent, OH 44242, USA
[#] Y.-S. Guan and J. Qiao contributed equally to this work.

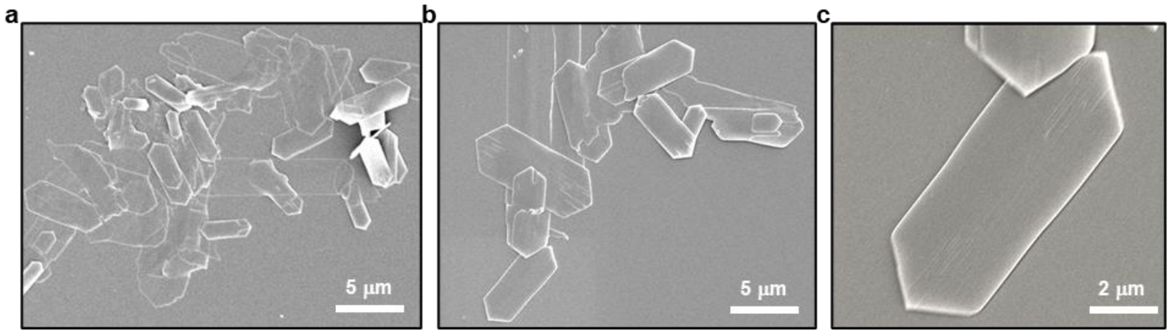

Figure S1. The SEM images of the TDPPQ assemblies fabricated from TDPPQ solutions with different concentrations. (a) 5 mg/mL (b) 1 mg/mL (c) 0.1 mg/mL.

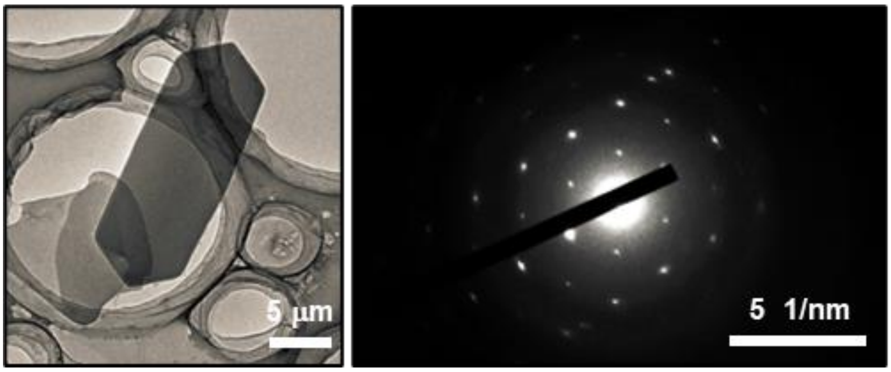

Figure S2. The TEM image of the TDPPQ nanosheet and corresponding selected area electron diffraction (SAED) pattern.

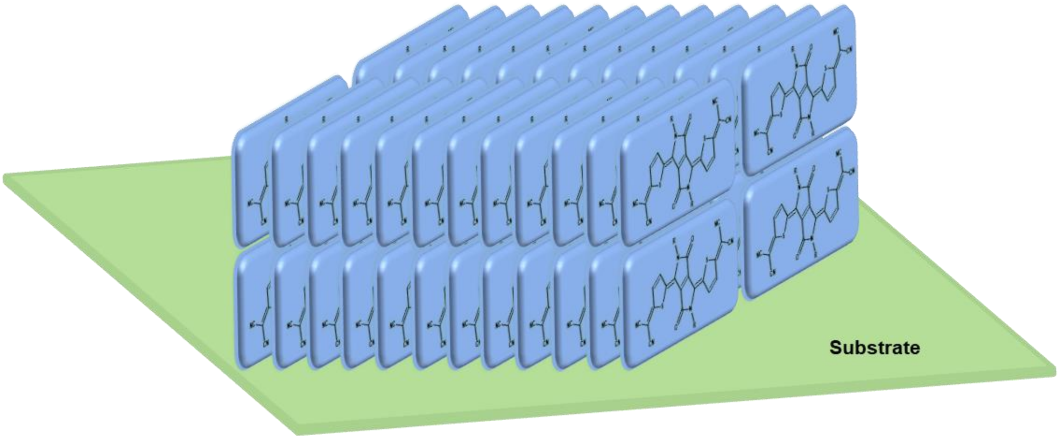

Figure S3. The packing structure of TDPPQ molecules within the TDPPQ nanosheets.

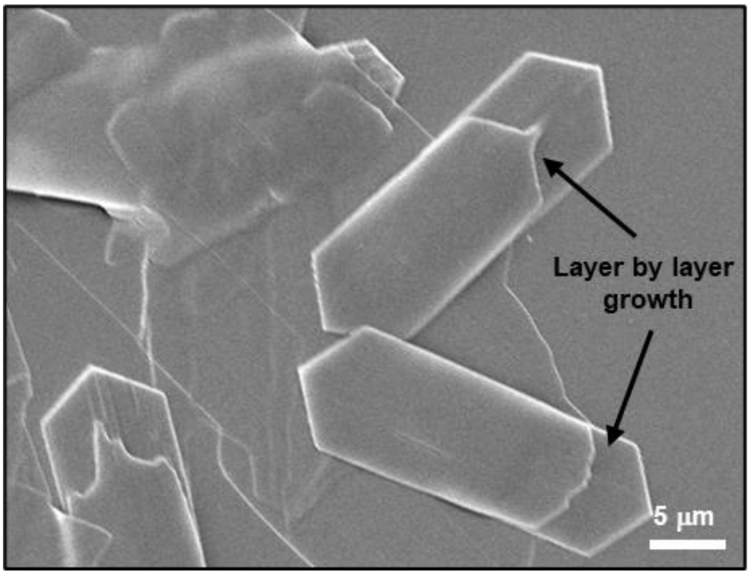

Figure S4. The SEM image of the TDPPQ nanosheets revealing the layer-by-layer growth of the TDPPQ molecules.

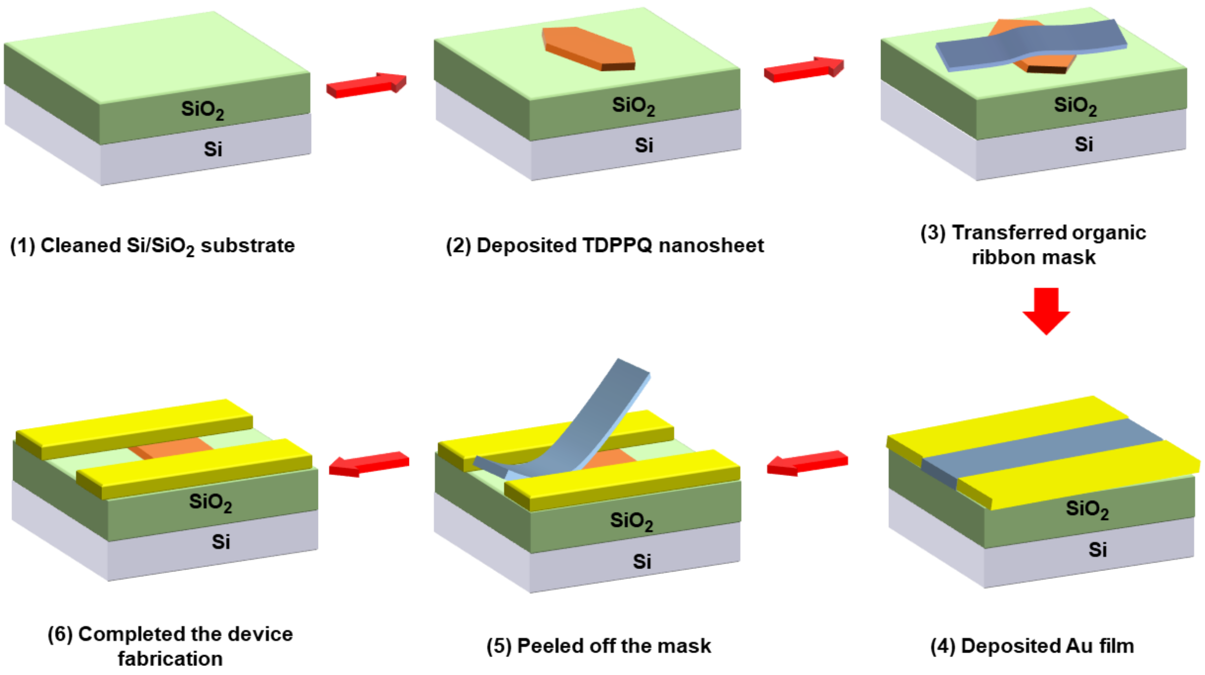

Figure S5. The schematic illustration of the device fabrication process.

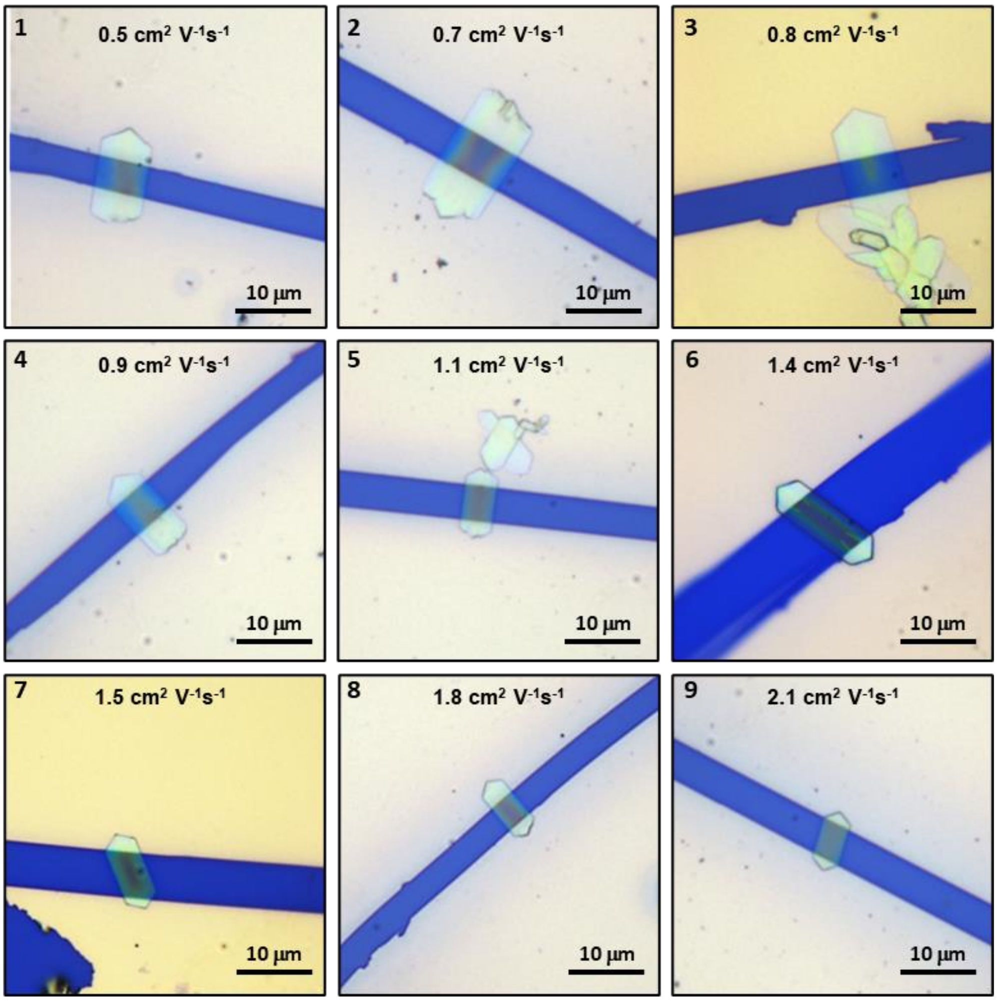

Figure S6. The optical image of the real TDPPQ nanosheet based organic transistor.

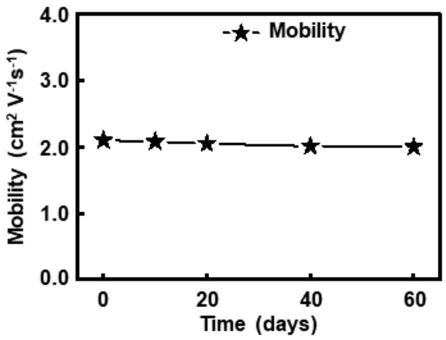

Figure S7. Stability of device under ambient conditions.

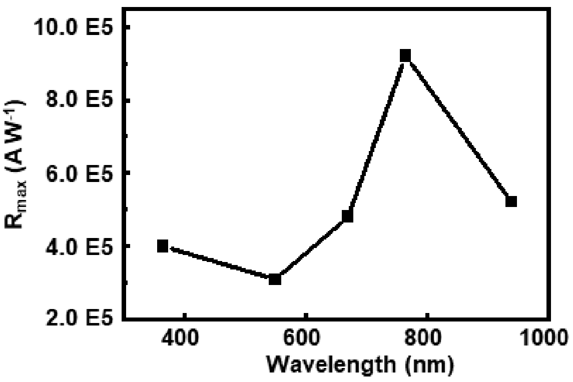

Figure S8. The maximum photoresponsivity (*R*) of the crystalline TDPPQ nanosheet based phototransistors at monochromatic light of different wavelength.

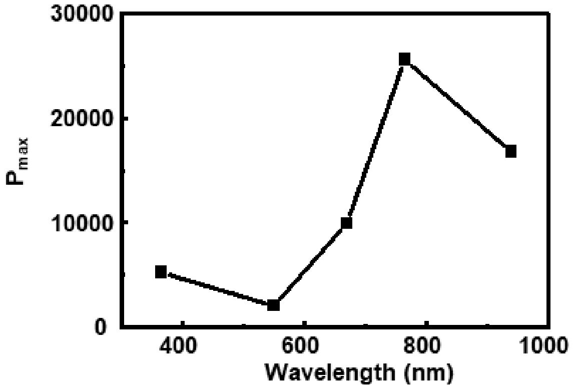

Figure S9. The photosensitivity (*P*) of the crystalline TDPPQ nanosheet based phototransistors at monochromatic light of different wavelength.

Table S1. The performance comparison of the NIR phototransistor based on organic semiconductors.

Materials	Mobility ($cm^2V^{-1}s^{-1}$)	R ($A W^{-1}$)	P	Ref.
TDPPQ	2.1	9.2×10^5 (940 nm)	2.5×10^4	This work
TFT-CN	1.36	9×10^4 (808 nm)	5×10^5	1
PIDTT-NDI	4.05×10^{-4}	8.42×10^{-3} (754 nm)	N/A	2
CuPc/PbPc: PTCDA	1.2×10^{-3}	0.322 (808 nm)	9.4×10^2	3
BODIPY-BF_2	1.13×10^{-1}	7.82×10^3 (960 nm)	1.18×10^3	4
PDIBDF-TT	5×10^{-3}	0.44 (808 nm)	3.3×10^4	5

References:

[1] C. Wang, X. Ren, C. Xu, B. Fu, R. Wang, X. Zhang, R. Li, H. Li, H. Dong, Y. Zhen, S. Lei, L. Jiang, W. Hu, *Adv. Mater.* **2018,** 30, e1706260.

[2] S. Lee, C. Lee, H. Kim, Y. Kim, *J. Mater. Chem. C* **2020,** 8, 15778.

[3] Y. Peng, W. Lv, B. Yao, G. Fan, D. Chen, P. Gao, M. Zhou, Y. Wang, *Org. Electron.* **2013,** 14, 1045.

[4] F. Li, Y. Chen, C. Ma, U. Buttner, K. Leo, T. Wu, *Adv. Electron. Mater.* **2017,** 3, 1600430.

[5] M. Zhu, S. Lv, Q. Wang, G. Zhang, H. Lu, L. Qiu, *Nanoscale* **2016,** 8, 7738.
